# Simeprevir and Sofosbuvir Combination Treatment in a Patient with HCV Cirrhosis and HbS Beta 0-Thalassemia: Efficacy and Safety despite Baseline Hyperbilirubinemia

**DOI:** 10.1155/2016/7635128

**Published:** 2016-03-02

**Authors:** Nikolaos Papadopoulos, Melanie Deutsch, Athanasios Georgalas, Helias Poulakidas, Lazaros Karnesis

**Affiliations:** ^1^First Department of Internal Medicine, 401 General Army Hospital of Athens, 11525 Athens, Greece; ^2^Second Department of Internal Medicine, Athens University Medical School, Hippokration Hospital of Athens, 11527 Athens, Greece; ^3^Hematology Department, 401 General Army Hospital of Athens, 11525 Athens, Greece

## Abstract

Hyperbilirubinemia is an adverse reaction of simeprevir (SMV). The majority of these patients were taking concurrent ribavirin presenting elevated unconjugated hyperbilirubinemia due to hemolysis. However, cases of hepatic failure with elevated bilirubin level have also been reported in patients with decompensated cirrhosis. We describe a 51-year-old female patient with HbS beta 0-thalassemia and recently diagnosed compensated cirrhosis due to chronic hepatitis C infection. Laboratory evaluation revealed total bilirubin: 2.7 mg/dL and serum HCV-RNA 137.204 IU/mL. HCV was genotyped as 4. A FibroScan revealed 35.3 kPa. She was considered as illegible for pegylated-interferon-free treatment with direct acting antivirals and a course with simeprevir and sofosbuvir (SOF) combination for twelve weeks was planned. Hyperbilirubinemia developed from the beginning with peak values during the 3rd month of treatment. However, no findings of liver decompensation were noticed. Hyperbilirubinemia was benign and fully reversible and our patient finally achieved sustained virological response 24 weeks after the end of treatment.

## 1. Introduction

HbS beta 0-thalassemia is a variant of Sickle Cell Disease (SCD) with similar signs and symptoms requiring frequent blood transfusions. Since hepatitis C virus (HCV) is the major cause of posttransfusion hepatitis, patients with SCD and variants are reported to present chronic hepatitis C infection (CHC) with a prevalence of 7–67% [1]. The risk of developing cirrhosis and end stage liver disease in these patients is obviously increased due to not only the concomitant hepatic iron overload but also the fact that antiviral treatment is often withheld because of the concern of worsening anemia caused by the classical pegylated IFN-*α* (PegIFN-*α*) and ribavirin (RBV) treatment [2]. The use of new interferon-free, direct acting antivirals (DAAs) and regimens seems ideal in this setting of patients and is now recommended by the European Association for the Study of the Liver (EASL) 2015 [3]. However none of the DAA studies included patients with hemoglobinopathies or has been much real life experience reported by now. Therefore we report a case of severe, but reversible, hyperbilirubinemia in a patient with HbS beta 0-thalassemia secondary to treatment with simeprevir (SMV) and sofosbuvir (SOF) for chronic hepatitis HCV.

## 2. Case Report

A 51-year-old female patient with HbS beta 0-thalassemia and recently diagnosed HCV infection was referred from the Hematology Department. The patient was transfusion dependent (2-3 times per year) up to the age of 15 years when splenectomy was performed. Since then she usually maintains a hematocrit (Ht) around 22–24% with no longer need for blood transfusions. Apart from blood transfusions, she had no other known risk factors for HCV. On physical examination, jaundice, clubbing, hepatomegaly, palmar erythema, and vascular spiders were present. Laboratory evaluation revealed Ht: 23.2%, WBC: 9.49 × 10^9^/L, PLT: 194 × 10^9^/L, and total bilirubin: 2.7 mg/dL (direct bilirubin 0.9 mg/dL). Serum HCV-RNA was detected as 137.204 IU/mL (low viral load) by PCR (Cobas AmpliPrep/Cobas TaqMan HCV Test) with lower detection level of 12 IU/mL. HCV was genotyped using the INNO-LIPA HCV II probe assay as 4 (Table 1). A FibroScan evaluation revealed 35.3 kPa (IQR: 3.3), and the APRI score was 1.75. Ultrasound and computer tomography (surface nodularity with increased echogenicity, hypertrophy of the caudate lobe and lateral segments of left lobe (segments II and III) with concomitant atrophy of the posterior segments (VI and VII) of the right lobe) were also compatible with compensated cirrhosis (Child-Pugh-Turcotte score: 6, class: A, MELD score: 11).

She was considered as illegible for IFN-free treatment with DAAs combination. The patient started the only DAAs combination available in Greece in June 2014: SOF 400 mg per day p.o. and SMV 150 mg per day p.o. for 12 weeks. Close monitoring (follow-up per week) was planned for her as well as HCV-RNA detection in week 4. Her other significant history revealed cholecystectomy and breast cancer for which radical mastectomy was performed 8 years earlier. Medications included folic acid. She had never smoked or drunk alcohol.

In the first scheduled follow-up, the patient appeared jaundiced with no other remarkable clinical findings. Laboratory evaluation revealed hyperbilirubinemia in the absence of hepatotoxicity as indicated by normal serum aminotransferases, alkaline phosphatase, and Prothrombin Time (PT) expressed as International Normalized Ratio (INR). We used the Naranjo probability scale to assess the probability of SMV adverse reaction. A total score value of six indicated SMV as a probable cause of hyperbilirubinemia [4]. The patient completed the 1st month of treatment with nondetectable HCV-RNA, while total bilirubin was as high as 4.74 mg/dL and direct bilirubin was 3.51 mg/dL. During the 2nd month of treatment serum total bilirubin rose to 7.76 mg/dL and remained stable-high during the 3rd month with no clinical or laboratory liver function deterioration. During treatment, Ht remained between 23.2 and 25.4% with no signs of hemolysis and no need for blood transfusion. The patient completed 12 weeks of treatment and her serum total bilirubin levels 6 months after stopping treatment reverted to those prior to treatment (2.76 mg/dL). Moreover she achieved nondetectable HCV-RNA (SVR).

## 3. Discussion

Patients with hemoglobinopathies are at high risk of HCV infection, particularly if transfused before the introduction of HCV donor screening programs. Despite improvements in blood transfusion policy and antiviral therapy in combination with iron chelation therapy, HCV related liver disease represents an important cause of mortality in these patients. Classical PegIFN/RBV combination represented until recently the only treatment option; however it was often withheld because of side effects, especially worsening preexisting anaemia [2]. In the new era of IFN-free HCV regimens, direct acting antivirals (DAAs) seem to be ideal candidates to control liver inflammation and to improve the prognosis in patients with CHC and hemoglobinopathies especially when they present with cirrhosis [3]. However there are very few data regarding their efficacy and safety in this setting of patients.

Our patient presented with HbS beta 0-thalassemia and compensated HCV cirrhosis genotype 4 and was successfully treated with SMV/SOF combination.

SMV in combination with SOF is currently approved for the treatment of cirrhotic patients with HCV genotype 1 infection [5]. No trials with this combination in genotype 4 population have been published until now, so still some therapeutic issues may be challenging. Nevertheless, given the effectiveness against genotype 4 of SOF or SMV in other combinations, it is likely that the results of the COSMOS trial in patients infected with genotype 1 could be also extrapolated in genotype 4 patients [3, 5].

One of the concerns regarding DAA treatment regimens is hyperbilirubinemia which was rarely recognized as a side effect of SOF but was a relatively common adverse reaction of SMV [6–8]. About 7% of the patients in the main registration studies presented hyperbilirubinemia, classified as grade 1 (≤1.5x ULN), grade 2 (≤2.5x ULN), grade 3 (≤5x ULN), and grade 4 (>5x ULN) [7]. Hyperbilirubinemia events were transient with peak values of 7–10 mg/dL at week 2 and resolved completely after the end of treatment [5]. The majority of these patients were taking concurrent RBV presenting especially elevated unconjugated hyperbilirubinemia due to hemolysis [9]. However, postmarketing cases of hepatic decompensation and hepatic failure with markedly elevated direct bilirubin level have also been reported in patients treated with SMV [8, 10]. The usual reported pattern of SMV hyperbilirubinemia was the mixed one (conjugated and unconjugated) and was possibly attributed to the inhibition of bilirubin hepatic transporters OATP1B1 and MRP2 by SMV resulting in the blockage of bilirubin clearance and drug-mediated reduction in hepatic uptake and/or conjugation of bilirubin, not associated with evidence of hepatotoxicity or hemolysis [11–13]. However, patients with advanced cirrhosis may have lower CYP3A activity leading them to impaired SMV metabolism and dose-dependent toxicity with acute on chronic liver failure [14]. Since most cases were reported in patients with advanced and/or decompensated cirrhosis, SMV is not recommended for patients with moderate or severe hepatic impairment [8].

Despite this knowledge, there are no recommendations regarding the use of SMV in patients who already present with elevated bilirubin at baseline. This is the case of the majority of the patients with hemoglobin abnormalities. Moreover, it is not clear how these patients should be followed during the treatment especially if they also present with cirrhosis.

Our cirrhotic patient was already icteric before treatment initiation. Peak hyperbilirubinemia developed during the 3rd month of treatment (>3x baseline levels) with no clinical signs or laboratory findings of liver injury. However SMV mediated hyperbilirubinemia was benign and fully reversible [15]. Moreover, our patient finally achieved an excellent outcome with SVR 24 weeks after the end of treatment and no signs of liver decompensation.

## 4. Conclusion

Our experience indicates that SMV, despite hyperbilirubinemia, is effective and safe in combination with SOF in compensated cirrhotic thalassemic patients.

## Figures and Tables

**Table 1:** Laboratory evaluation prior to and during treatment.

	Initial	1st month	2nd month	3rd month	6 months after treatment
HCV-RNA, IU/mL	137.204		Nondetectable		Nondetectable
Genotype	4				
Ht, %	23.6	23.2	24.6	25.4	26
Total bilirubin, mg/dL	2.3	4.74	7.76	7.78	2.76
Direct bilirubin, mg/dL	1.2	3.51	3.54	3.6	0.9
ALT, U/L	66	33	38	36	36
Alkaline phosphatase, U/L	115	143	125	143	120
Albumin, g/dL	3.8	3.7	3.8	3.7	3.8
International Normalized Ratio (INR)	1.1	1.1	1.1	1.1	1.1
